# Color Photometric Stereo Using Multi-Band Camera Constrained by Median Filter and Occluding Boundary

**DOI:** 10.3390/jimaging5070064

**Published:** 2019-07-16

**Authors:** Daisuke Miyazaki, Yuka Onishi, Shinsaku Hiura

**Affiliations:** 1 Graduate School of Information Sciences, Hiroshima City University, Hiroshima 731-3194, Japan; 2 Graduate School of Engineering, University of Hyogo, Hyogo 671-2280, Japan

**Keywords:** photometric stereo, color photometric stereo, multispectral imaging

## Abstract

One of the main problems faced by the photometric stereo method is that several measurements are required, as this method needs illumination from light sources from different directions. A solution to this problem is the color photometric stereo method, which conducts one-shot measurements by simultaneously illuminating lights of different wavelengths. However, the classic color photometric stereo method only allows measurements of white objects, while a surface-normal estimation of a multicolored object using this method is theoretically impossible. Therefore, it is necessary to add some constraints to estimate the surface normal of a multicolored object using the framework of the color photometric stereo method. In this study, a median filter is employed as the constraint condition of albedo, and the surface normal of the occluding boundary is employed as the constraint condition of the surface normal. By employing a median filter as the constraint condition, the smooth distribution of the albedo and normal is calculated while the sharp features at the boundary of different albedos and normals are preserved. The surface normal at the occluding boundary is propagated into the inner part of the object region, and forms the abstract shape of the object. Such a surface normal gives a great clue to be used as an initial guess to the surface normal. To demonstrate the effectiveness of this study, a measurement device that can realize the multispectral photometric stereo method with seven colors is employed instead of the classic color photometric stereo method with three colors.

## 1. Introduction

To reproduce a detailed surface shape, normal information is necessary. To obtain this information, the photometric stereo method was proposed, which estimates the normal by transitioning the brightness levels of several pictures by changing the direction of the light source. However, as it requires multiple photoshoots, the photometric stereo method is not suitable for modeling a moving object. To measure the shape of a moving object, the color photometric stereo method, which employs several colored light sources, was developed. This method involves placing light sources of red, green, and blue colors in three different directions, which simultaneously illuminate the target object. This paper proposes a technique that employs some constraints so that it can be applied to colored objects, which is impossible for conventional color photometric stereo. Unlike the common color photometric stereo method, we use seven narrow-band lights with different peak wavelengths while observing the target object with a seven-band multispectral camera.

## 2. Related Work

The photometric stereo method [1,2] estimates the normal of the surface of an object by illuminating the object and analyzing the resulting shadings on the object’s surface. In this method, light is illuminated on the object from one white parallel light source (an infinity point light source) to obtain a picture. Then, two more pictures are captured with different light source directions. In other words, it requires capturing three pictures with different light source directions. Therefore, it is impossible to measure a dynamic object. This problem can be resolved using the color photometric stereo method. In this method, lights are simultaneously illuminated from red, green, and blue light sources, and one picture photographed with an RGB color camera is captured. Such one-shot photograph enables the measurement of a dynamic object.

The color photometric stereo method [3,4,5] (also known as shape-from-color) was developed in the 1990s. Since then, various studies [4,6,7,8,9,10,11,12,13,14,15,16,17,18,19,20,21,22] have been conducted in this regard. However, many problems are inherent in the color photometric stereo method. Many researchers in the past have struggled with this method, and even until recently it has been an ongoing problem. The principle problem of the color photometric stereo method is the fact that it can only be used with white objects. This is an inevitable problem as long as lights are illuminated from three colored light sources to estimate the surface normal.

Recently, various techniques have been proposed to apply the color photometric stereo method to multicolored objects. Roubtsova et al. [20] applied the color photometric stereo method to objects with arbitrary BRDF (bidirectional reflectance distribution function) by incorporating the Helmholtz Stereo method. However, the principle of this method does not allow for real-time measurement. Therefore, an optical flow is required to measure a dynamic object. Kim et al. [16] and Gotardo et al. [23] also tracked dynamic objects using optical flow, and estimated the surface shape of objects by utilizing several images taken at different times. Fyffe et al. [12] proposed a color photometric stereo method that employs six band cameras and three white color sources. All three light sources used in their method appear white to the human eye. However, all of them possess different spectral distributions. Furthermore, this method pre-measures the reflectance of various objects to prepare a database, and calculated four bases. Using this technique, it is possible to obtain an analytic solution, as there are four unknown numbers in relation to albedo (four base coefficients) and two in relation to the normal (because the three-dimensional vector is normalized), and six equations are obtainable. Anderson et al. [6] estimated the object color using the normal of multi-view stereo. However, owing to the low accuracy of the normal of multi-view stereo, they improved the estimation accuracy of object color based on the hypothesis that an object is composed of a limited number of colors. Their technique incorporates the framework of region segmentation, where the number of the regions is automatically determined based on the Bayesian information criterion. Chakrabarti et al. [8] calculated the candidates of object color by approximating the shape inside the patch of neighboring areas using a polynomial. They calculated the histogram of the object color candidates, chose only the limited number of colors that gained most votes, and evaluated the normal by postulating that the object is composed of these limited number of colors. Jiao et al. [15] divided a picture into super pixel regions and estimated the normal by postulating that the object color inside each region is uniform.

In this paper, the problem faced by the color photometric stereo method is solved using a different approach from those used in previous studies. Our proposed technique employs a median filter as the constraint condition of the albedo and surface normal. We also use the occluding boundary constraint for the surface normal. Thanks to this constraint, we have a good estimate from the initial state of surface normal, which results in robust estimation.

The techniques of Gotardo et al. [23], Kim et al. [16], and Roubtsova et al. [20] need to employ optical flow to measure a dynamic object, while the technique of Fyffe et al. [12] requires a reflectance database to be prepared prior to the measurement. Our proposed technique does not require a shape obtained from other sensors such as multi-view stereo or laser sensor, unlike the technique of Anderson et al. [6] Moreover, unlike the techniques of Chakrabarti et al. [8] and Jiao et al. [15] our proposed method does not require region segmentation. Previous color photometric stereo methods used three lights with red, green, and blue colors and observed the object with an RGB color camera. In our study, seven lights with different wavelengths are used to illuminate the object, which is then observed by a seven-band multispectral camera. This paper demonstrates that multi-spectral cameras and multi-spectral light sources are also effective for the color photometric stereo method.

## 3. Multispectral Color Photometric Stereo Method

### 3.1. Image Formulation

A photometric stereo method that employs independent colored light is called the color photometric stereo method. A characteristic of this method is that it enables the estimation of the surface normal with one photoshoot. The widespread color photometric stereo method is conducted with three types of colored lights. While the conventional photometric stereo method results in several grayscale images, the color photometric stereo method results in a multi-spectral image.

Although the fundamental theory is given in several sources in the literature [24,25], we briefly explain the formulation of the problem. The spectral sensitivity of a camera is denoted as $Q_{c}\left(\lambda \right)$, the spectral distribution of the light source is E(λ), and the spectral reflectance of the object is S(λ). Moreover, *c* denotes the channel. In this case, the brightness obtained from each channel of the camera can be attained from Equation (1).

(1) $$I_{c}=\int_{0}^{\infty }Q_{c}\left(\lambda \right)E\left(\lambda \right)S\left(\lambda \right)d\lambda \;.$$

Suppose that we use single light E(λ) whose spectral distribution can be represented as a delta function δ(·) whose peak wavelength is $\lambda_{c}$.

(2) $$E\left(\lambda \right)=e_{c}\delta \left(\lambda -\lambda_{c}\right)\;,$$

where $e_{c}$ represents the brightness of the light. Suppose that the channel *c* is only sensitive to the wavelength $\lambda_{c}$, and suppose that other channels cannot detect the wavelength $\lambda_{c}$.

(3) $$Q_{c}\left(\lambda \right)E\left(\lambda \right)=q_{c}e_{c}\delta \left(\lambda -\lambda_{c}\right)\;,$$

where $q_{c}$ represents the sensitivity at wavelength $\lambda_{c}$. Suppose that we lit a single parallel light source (infinite-far point light source) whose spectral distribution is represented as delta function and its peak wavelength is $\lambda_{c}$, the pixel brightness $I_{c}$ can be represented as follows using the formulation that the diffuse reflection is represented as $S\left(\lambda_{c}\right)={\tilde{s}}_{c}\max\left(n\cdot l_{c},0\right)$.

(4) $$I_{c}=q_{c}e_{c}{\tilde{s}}_{c}\max\left(n\cdot l_{c},0\right)\;,$$

where ${\tilde{s}}_{c}$ represents the reflectance. n is a normal vector and $l_{c}$ is the light source direction vector of channel *c*. Denoting as $A_{c}=q_{c}e_{c}{\tilde{s}}_{c}$, Equation (4) becomes as follows.

(5) $$I_{c}=A_{c}\max\left(n\cdot l_{c},0\right)\;.$$

Hereinafter, we call $A_{c}$ albedo. Note that the camera sensitivity and light source brightness are included in $A_{c}$.

**Figure 1:**
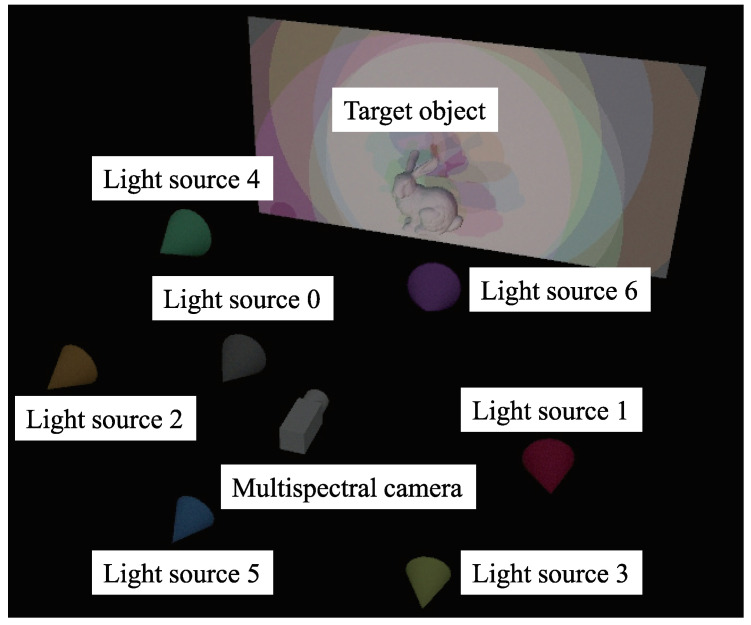
Conceptual explanation of multispectral color photometric stereo. Target object is illuminated by multiple light sources whose wavelengths are different. One image is taken using multispectral camera.

**Figure 2:**
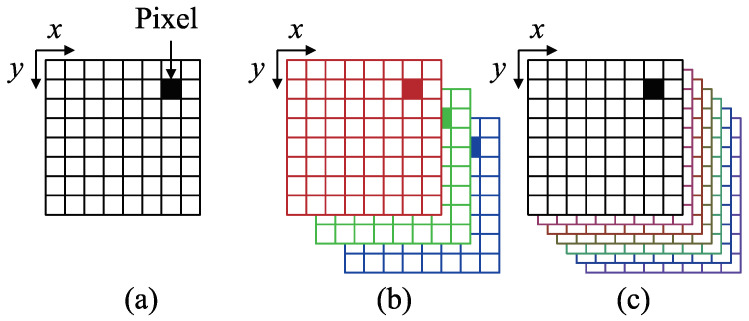
Explanation of multi-channel image: (**a**) Grayscale image with single channel, (**b**) RGB color image with 3 channels, and (**c**) multispectral image with 7 channels.

As shown in Figure 1, this study conducts a photoshoot of a multicolored object using seven channels (Figure 2). Following Equation (5), the brightness is obtained from this photoshoot as follows.

(6) $$\begin{array}{lll} I_{0} & = & A_{0}\max\left(n\cdot l_{0},0\right)\;,\\ I_{1} & = & A_{1}\max\left(n\cdot l_{1},0\right)\;,\\ & \vdots & \\ I_{6} & = & A_{6}\max\left(n\cdot l_{6},0\right)\;. \end{array}$$

The surface normal n is a 3D vector; however, the degree-of-freedom is two because it is constrained to be a unit vector (such constraint reduces one degree-of-freedom). Albedo $A_{c}$ is represented by seven parameters. There are seven equations, as shown in Equation (6), and nine unknown parameters ($A_{0}$, $A_{1}$, …, $A_{6}$, $n_{x}$, $n_{y}$, $n_{z}$, s.t., $n_{x}^{2}+n_{y}^{2}+n_{z}^{2}=1$, namely seven for albedo and two for surface normal). Therefore, color photometric stereo, without any assumption or constraint, is an ill-posed problem.

The most commonly used assumption is to limit the color of the target objects to white ($A_{0}=A_{1}=\cdots =A_{6}$). If we set $s=A_{c}n$ and if we ignore the shadow, the surface normal s (scaled with albedo) can be directly solved.

(7) $$\left(\begin{array}{c} s \end{array}\right)={\left(\begin{array}{c} l_{0}^{⊤}\\ l_{1}^{⊤}\\ \vdots \\ l_{6}^{⊤} \end{array}\right)}^{+}\left(\begin{array}{c} I_{0}\\ I_{1}\\ \vdots \\ I_{6} \end{array}\right)\;.$$

As is shown above, the color photometric stereo for white objects, or in other words, the conventional photometric stereo can directly solve the surface normal, without iterative optimization nor additional constraints such as smoothness constraints. However, this paper analyzes the methods with multi-colored objects. Therefore, we add smoothness constraints and iteratively solved the problem formulated as Equation (6).

The proposed technique estimates the surface normal through an iteration process. The cost function that is minimized through the iteration process is explained in Section 3.2. Each term of the cost function is explained in Section 3.3, Section 3.4, Section 3.5 and Section 3.6. The initial value required in the iteration process is explained in Section 3.6 and Section 3.7, and the update rule for each iteration is shown in Section 3.8. Detection of specular reflection is explained in Section 3.9. A method to integrate the surface normal to obtain the geometrical structure of the object surface is shown in Section 3.10, and Section 3.11 explains how to cancel the channel crosstalk.

### 3.2. Cost Function

The cost function ∫∫Fdxdy is expressed through the following four terms:

(8) $$\begin{array}{ll} F= & \int \;\;\int_{(x,y)\in P\backslash \partial P}F_{1}\left(n\left(x,y\right),A\left(x,y\right),I\left(x,y\right),L\right)dxdy\\ & +\int \;\;\int_{(x,y)\in P\backslash \partial P}F_{2}\left(n\left(x,y\right)\right)dxdy\\ & +K_{2}\int \;\;\int_{(x,y)\in P\backslash \partial P}F_{3}\left(A\left(x,y\right)\right)dxdy\\ & +\int \;\;\int_{(x,y)\in \partial P}F_{4}\left(n\left(x,y\right)\right)dxdy\;. \end{array}$$

Equation (8) is minimized under the condition that surface normal n should be an unit vector, ∥n∥=1. Here, $A={\left(A_{0}\left(x,y\right),A_{1}\left(x,y\right),\cdots ,A_{6}\left(x,y\right)\right)}^{⊤}$, $L=(l_{0},l_{1},$ $\cdots ,l_{6}{)}^{⊤}$, and $I={\left(I_{0}\left(x,y\right),I_{1}\left(x,y\right),\cdots ,I_{6}\left(x,y\right)\right)}^{T}$. $K_{2}$ is the Lagrange multiplier. The area where the target object is observed is denoted as P, and the occluding boundary is denoted as ∂P. The first three terms $F_{1}$, $F_{2}$, and $F_{3}$ are the soft constraints defined inside the object region P\∂P, and the fourth term $F_{4}$ is the hard constraint defined at the occluding boundary ∂P. Orthographic projection is assumed in this paper for camera model.

Following are the four terms of cost functions, where $K_{11}$ and $K_{12}$ are the Lagrange multipliers.

(9) $$\begin{array}{ccc} F_{1} & = & \sum_{c=0}^{6}{\left(I_{c}\left(x,y\right)-A_{c}\left(x,y\right)\max\left(l_{c}^{T}n\left(x,y\right),0\right)\right)}^{2}\;, \end{array}$$

(10) $$\begin{array}{ccc} F_{2} & = & K_{11}\left({∥\frac{\partial n(x,y)}{\partial x}∥}^{2}+{∥\frac{\partial n(x,y)}{\partial y}∥}^{2}\right)+K_{12}\left(∥\frac{\partial n(x,y)}{\partial x}∥+∥\frac{\partial n(x,y)}{\partial y}∥\right)\;, \end{array}$$

(11) $$\begin{array}{ccc} F_{3} & = & ∥\frac{\partial A(x,y)}{\partial x}∥+∥\frac{\partial A(x,y)}{\partial y}∥\;, \end{array}$$

(12) $$\begin{array}{ccc} F_{4} & = & {\left|\left|n\left(x,y\right)-n_{b}\left(x,y\right)\right|\right|}^{2}\;. \end{array}$$

Section 3.3, Section 3.4, Section 3.5 and Section 3.6 explain $F_{1}$, $F_{2}$, $F_{3}$, and $F_{4}$, respectively. $F_{1}$ expresses the residual of Lambertian reflectance and the input image brightness. *I* is the input image brightness, *A* is the albedo, l is the light source direction, and n is the surface normal. Here, *c* is the index that identifies the channel, and $\max(l^{⊤}n,0)$ represents the shading. $F_{2}$ is the smoothing term of the surface normal, and indicates that the gradient of the surface normal should be small; $F_{3}$ is the smoothing term of albedo, and indicates that the gradient of albedo should be small; and $F_{4}$ is the constraint condition of the surface normal at the occluding boundary. The surface normal at the occluding boundary $n_{b}$ can be derived from differential geometry. $F_{4}$ indicates that the surface normal at the occluding boundary should be equal to $n_{b}$. The reason why $F_{2}$ use both L1 norm and L2 norm is discussed in Section 3.4.

As we will explain in Section 3.3, Section 3.4, Section 3.5 and Section 3.6, we do not minimize Equation (8) at once but minimize $F_{1}$, $F_{2}$, $F_{3}$, and $F_{4}$ separately. Although we cannot mathematically prove that such piecewise minimization results in global minimum, it is empirically known that piecewise minimization make the cost function smaller through the iteration. Since Equation (8) is a non-linear equation with several number of constraints, convergence speed is low. On the other hand, our approach is robust, stable, and speedy since we can minimize the cost function with closed form solution as is shown in Section 3.3 and Section 3.6 ($F_{1}$ and $F_{4}$) and minimizing it with straightforward filtering as is shown in Section 3.4 and Section 3.5.

Section 3.3 explains that $F_{1}$ solely cannot solve the problem. In order to solve the problem, we have to add $F_{2}$ or $F_{3}$ as it will be explained in Section 3.4 and Section 3.5. The surface normal will be smooth if we add $F_{2}$, and the albedo will be smooth if we add $F_{3}$. If we add both $F_{2}$ and $F_{3}$, the surface normal and the albedo becomes relatively sharper than adding either $F_{2}$ or $F_{3}$. Since we want to suppress the surface normal and the albedo to be smooth, we add not only $F_{2}$ and $F_{3}$ but also $F_{4}$.

### 3.3. Determining Surface Normal and Albedo

If we ignore the influence of the shadow, the first term $F_{1}$ shown in Equation (9) can be represented as Equation (13).

(13) $$F_{1}=\sum_{c=0}^{6}{\left(I_{c}\left(x,y\right)-A_{c}\left(x,y\right)\left(l_{c}^{⊤}n\left(x,y\right)\right)\right)}^{2}\;.$$

The solution obtained by minimizing Equation (13) is expressed as Equation (14).

(14) $$I_{c}\left(x,y\right)=A_{c}\left(x,y\right)\left(l_{c}^{⊤}n\left(x,y\right)\right)\;.$$

When albedos $A_{0},A_{1},\cdots ,A_{6}$ are known, the surface normal n can be obtained by calculating the pseudo-inverse matrix $L^{+}$ of matrix L, as shown in Equation (15).

(15) $$\left(\begin{array}{c} n_{x}\\ n_{y}\\ n_{z} \end{array}\right)={\left(\begin{array}{ccc} l_{x0} & l_{y0} & l_{z0}\\ l_{x1} & l_{y1} & l_{z1}\\ & \vdots & \\ l_{x6} & l_{y6} & l_{z6} \end{array}\right)}^{+}\left(\begin{array}{c} I_{0}\left(x,y\right)/\left(A_{0}\left(x,y\right)+\varepsilon_{1}\right)\\ I_{1}\left(x,y\right)/\left(A_{1}\left(x,y\right)+\varepsilon_{1}\right)\\ \vdots \\ I_{6}\left(x,y\right)/\left(A_{6}\left(x,y\right)+\varepsilon_{1}\right) \end{array}\right)\;.$$

Here, $\varepsilon_{1}$ is a small positive constant introduced to prevent division-by-zero. As the surface normal n is expressed as a unit vector (∥n∥=1), it is normalized after calculating Equation (15). The unit vector $\hat{n}$ of the surface normal n can be calculated by dividing its length ∥n∥ as $\hat{n}=n/∥n∥$.

Shadow has a low brightness, and thus, thresholding the brightness results in detecting the shadow, as is shown in Section 3.9. As for the channel which is detected as a shadow using the procedure shown in Section 3.9, Equation (15) cannot be used for surface normal estimation. To avoid this, n is calculated by weighting the *c*’th row of L by a small value *d* in relation to channel *c*, which is a shadow. This situation is expressed as follows.

(16) $$\left(\begin{array}{c} n_{x}\\ n_{y}\\ n_{z} \end{array}\right)={\left(\begin{array}{ccc} l_{x0} & l_{y0} & l_{z0}\\ \vdots & \vdots & \vdots \\ l_{x,c-1} & l_{y,c-1} & l_{z,c-1}\\ dl_{x,c} & dl_{y,c} & dl_{z,c}\\ l_{x,c+1} & l_{y,c+1} & l_{z,c+1}\\ \vdots & \vdots & \vdots \\ l_{x6} & l_{y6} & l_{z6} \end{array}\right)}^{+}\left(\begin{array}{c} I_{0}\left(x,y\right)/\left(A_{0}\left(x,y\right)+\varepsilon_{1}\right)\\ \vdots \\ I_{c-1}\left(x,y\right)/\left(A_{c-1}\left(x,y\right)+\varepsilon_{1}\right)\\ dI_{c}\left(x,y\right)/\left(A_{c}\left(x,y\right)+\varepsilon_{1}\right)\\ I_{c+1}\left(x,y\right)/\left(A_{c+1}\left(x,y\right)+\varepsilon_{1}\right)\\ \vdots \\ I_{6}\left(x,y\right)/\left(A_{6}\left(x,y\right)+\varepsilon_{1}\right) \end{array}\right)\;.$$

As usual, the surface normal n is normalized after calculating Equation (16).

When the surface normal is known, albedo can be calculated as shown in Equation (17) derived from Equation (14).

(17) $$A_{c}=\frac{I_{c}}{l_{c}^{⊤}n}\;.$$

To prevent division-by-zero, Equation (17) is calculated when $l_{c}^{⊤}n>\varepsilon_{2}$ holds, where $\varepsilon_{2}$ is a small positive constant. In addition, if the pixel is detected as an outlier (Section 3.9), Equation (17) is also not calculated.

There are seven constraint condition equations in Equation (14). The input brightness $I_{0},I_{1},\cdots ,I_{6}$ and the unit vector that expresses the light source directions $l_{0},l_{1},\cdots ,l_{6}$ are known. Albedos $A_{0},A_{1},\cdots ,A_{6}$ and normal vectors $n_{x},n_{y},n_{z}$ are unknown parameters. Because the 3D normal vector is conditioned to be the unit vector, its degree-of-freedom is two. Therefore, the total number of unknown parameters is nine, with seven albedos and two surface normal components. At this point, calculations are not possible because the number of the unknown numbers is larger than the number of equations. Thus, the smoothing of the surface normal, smoothing of albedos, and constraint condition of the surface normal at the occluding boundary are introduced to the cost function.

### 3.4. Smoothness Constraint for Surface Normal

As explained in Section 3.3, surface normal and albedo cannot be calculated because there are too many unknowns. Therefore, the smoothing of the surface normal is conducted as a constraint condition. The second term $F_{2}$ of cost function *F*, which expresses the smoothing term of the normal, is expressed as Equation (10).

The discretization of the first term of Equation (10) results in Equation (18) and that of the second term results in Equation (19).

(18) $$\begin{array}{ccc} n(x,y) & = & \frac{1}{4}\left\{n\left(x+1,y\right)+n\left(x-1,y\right)+n\left(x,y+1\right)+n\left(x,y-1\right)\right\}\;, \end{array}$$

(19) $$\begin{array}{ccc} n(x,y) & = & \mathrm{median}\{n(x+1,y),n(x-1,y),n(x,y+1),n(x,y-1)\}\;. \end{array}$$

In our software, Equation (18) is implemented as a Gaussian filter, and Equation (19) is implemented as a median filter. Convolving Equation (18) multiple times can be approximated by a Gaussian filter. Therefore, instead of applying Equation (18) multiple times, we applied a Gaussian filter once. We first apply a median filter before the Gaussian filter. After the surface normal is smoothed, it is normalized to be a unit vector.

The fastest way to calculate Equation (19) is to calculate the median for each element as follows.

(20) $$\begin{array}{lll} n_{x} & = & \mathrm{median}\{n_{x}\left(x+1,y\right),n_{x}\left(x-1,y\right),n_{x}\left(x,y+1\right),n_{x}\left(x,y-1\right)\}\;,\\ n_{y} & = & \mathrm{median}\{n_{y}\left(x+1,y\right),n_{y}\left(x-1,y\right),n_{y}\left(x,y+1\right),n_{y}\left(x,y-1\right)\}\;,\\ n_{z} & = & \mathrm{median}\{n_{z}\left(x+1,y\right),n_{z}\left(x-1,y\right),n_{z}\left(x,y+1\right),n_{z}\left(x,y-1\right)\}\;. \end{array}$$

After that the vector is normalized to be a unit vector. This procedure calculates the median in Euclidean distance, not in Riemannian distance (geodesic distance). However, this difference does not matter in practice since the surface is assumed to be smooth: namely, since the angle between neighboring pixels is small, the Euclidean distance of two vectors can be approximated as the Riemannian distance.

In order to keep the sharp feature of surface normal, median filter (Equation (19)) is used. The median filter will not change surface normal over neighboring pixels at sharp features. Although a median filter is preferable to keep the sharp features, we also use a Gaussian filter (Equation (18)) to constrain the surface normal to be smooth. The median filter does not change the surface normal at shape features, and such pixels may be stuck in local minima. The Gaussian filter (Equation (18)) can modify the surface normal even for such edges. We empirically found beneficial to use both a median filter and Gaussian filter since these filters can find a good balance between smooth normals and sharp features.

As shown in Equation (6), there are nine unknown parameters and seven equations. Although Equation (18) or Equation (19) comprises three equations, the surface normal should be constrained as a unit vector; thus, Equation (18) or Equation (19) has two degrees-of-freedom. Now, we have nine unknown parameters and nine equations per pixel. The problem is now well-posed, but an over-smoothed surface normal will be obtained if we solely use this constraint. We add another constraint $F_{3}$, as shown in Section 3.5, in order to relatively reduce the influence of $F_{2}$.

### 3.5. Smoothness Constraint for Albedo

As discussed in Section 3.4, smoothing of the surface normal alone is insufficient as a constraint condition. Therefore, albedo smoothing is also conducted. The third term $F_{3}$ in the cost function, which expresses the albedo smoothing, is shown in Equation (11). Equation (11) is discretized as Equation (21).

(21) A(x,y)=median{A(x+1,y),A(x−1,y),A(x,y+1),A(x,y−1)}.

Namely, we applied median filter to the albedo. As shown in Equation (6), there are nine unknown parameters and seven equations. Equation (21) implies seven equations because there are seven channels. Now, we have 9 unknown parameters and 14 equations per pixel, which results in a well-posed problem. However, an over-smoothed albedo will be obtained if we solely use this constraint. We add another constraint $F_{2}$ as shown in Section 3.4 in order to relatively reduce the influence of $F_{3}$.

### 3.6. Occluding Boundary Constraint and Initial Value of Surface Normal

The target objects of this study are smooth and closed surfaces. Here, the occluding boundary is the border region where the surface normal of the object begins to turn toward the rear just before it becomes invisible. The angle between the observation direction vector and the normal vector is $90^{\circ }$ since we assume orthographic projection for camera model. It means that it is possible to correctly estimate the surface normal at the occluding boundary, which is orthogonal to the object area contour. This is incorporated into the cost function as $F_{4}$. The occluding boundary normal is defined as $n_{b}$ (Equation (12)). Now, the solution that minimizes $F_{4}$ is $n\left(x,y\right)=n_{b}\left(x,y\right)$. At the occluding boundary, $n_{b}$ is used as the estimation of the surface normal.

Although $F_{2}$ or $F_{3}$ are enough for solving Equation (8), also using $F_{4}$ is beneficial. The function $F_{2}$ itself has no boundary condition, and if we minimize $F_{2}$ only the surface normal will be extraordinarily smooth. In order to restrict the surface normal to be smooth, we will add $F_{4}$ as the boundary condition.

In addition, the pixel brightness close to the occluding boundary is unreliable, since it contains shadow in most of the channels. Since the reliability of the data term $F_{1}$ is small at the occluding boundary, adding $F_{4}$ is reasonable.

**Figure 3:**
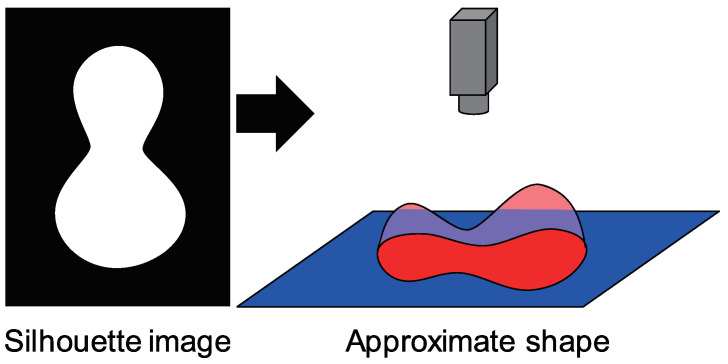
Approximate shape used for initial guess to surface normal. The shape is inflated using the silhouette of the object region.

To conduct the iteration process using the cost function, initial values are required for the surface normal and albedo. As follows, the initial value of surface normal is calculated from the surface normal at the occluding boundary (Figure 3). As is done in previous work [26], we also inflated the silhouette to make the approximate shape. Our approach is shown as follows.

First, we calculate the distance from each pixel to the nearest occluding boundary pixel, and next, we sort the distance. As for initial guess, we assume that the probability distribution of the height of the target object is the same as that of the hemisphere. Let us denote the maximum of the distance as $D_{\max}$. The number of the pixels in object region is |P|. The order of the sorted pixel (x,y) is denoted as *o*. If we assume that the object is hemisphere whose radius is *r*, then *r* is calculated from $\left|P\right|=\pi r^{2}$. The area *o* whose length from the center of the circle is denoted as *l* can be represented as $o=\pi l^{2}$. Therefore, *l* can be calculated from *o*. The height *h* is represented as $r^{2}=h^{2}+l^{2}$ where the distance from the center of the circle is *l*. Therefore, *h* can be calculated. Height of the unit hemisphere is calculated by dividing *r* from *h*. Multiplying $D_{\max}$ results in the height of the hemisphere where its maximum height is $D_{\max}$. After that, the height field is slightly smoothed.

The initial height (Figure 3) is obtained by above procedure. Differentiating the height and normalizing it as follows results in the surface normal $\hat{n}$.

(22) $$n_{x}=-\frac{\partial h}{\partial x}\;,\;n_{y}=-\frac{\partial h}{\partial y}\;,\;n_{z}=1\;.$$

(23) $$\hat{n}=\frac{\left(n_{x},n_{y},n_{z}\right)}{\sqrt{n_{x}^{2}+n_{y}^{2}+n_{z}^{2}}}\;.$$

Finally, the smoothed and normalized surface normal is used as the initial value.

### 3.7. Initial Value of Albedo

It is better to use an initial value of albedo which is close to the true albedo as much as possible, in order to speed up the convergence. However, since we do not use additional sensors or data, we have to calculate the initial albedo solely from input image. The input image is a single seven-channel image, whose light source direction is different. We calculate the average of seven channels, and this average image $I_{\mathrm{avg}}$ works well for initial albedo.

(24) $${\tilde{I}}_{\mathrm{avg}}\left(x,y\right)=\frac{1}{7}\left(I_{0}\left(x,y\right)+I_{1}\left(x,y\right)+\cdots +I_{6}\left(x,y\right)\right)\;,$$

(25) $$I_{\mathrm{avg}}=\mathrm{bilateral}\left({\tilde{I}}_{\mathrm{avg}}\right)\;.$$

**Figure 4:**
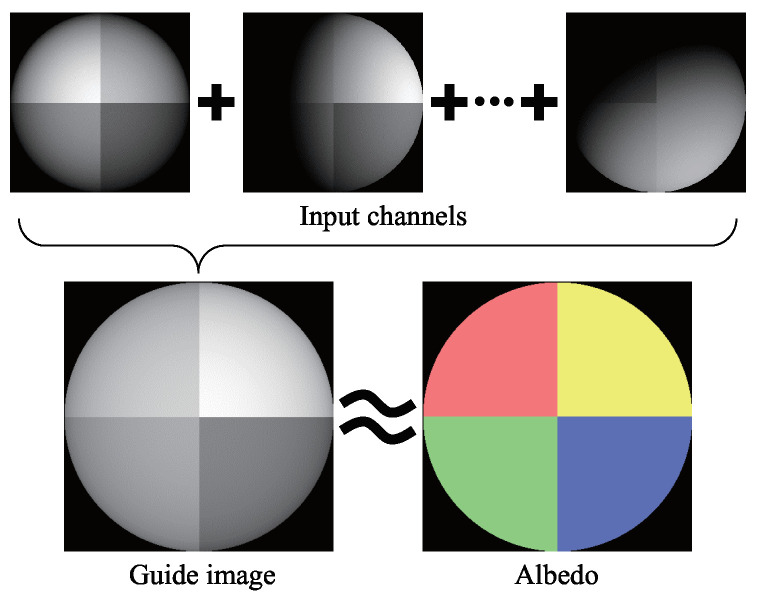
Average image calculated from seven channel images resembles the albedo.

This is the sole image we can obtain from seven input images closest to the true albedo. If an infinite number of light sources illuminate the object uniformly from the surroundings, the observation of the object becomes the same as that of the albedo with constant scaling. This is the reason why the average image can be a good estimate of albedo. As shown in Figure 4, the true albedo value and brightness of the average image $I_{\mathrm{avg}}$ are similar; therefore, the average image can be used as the initial guess. In order to decrease the effect of the shadow, the bilateral filter is applied to the average image.

The albedo A is highly correlated with the input image brightness I. The initial albedo $A_{c}\left(x,y\right)$ is set to be an image where the brightness of the average image $I_{\mathrm{avg}}$ is scaled to be the same as the brightness of each channel.

(26) $$A_{c}\left(x,y\right)=I_{\mathrm{avg}}\left(x,y\right)\underset{(\tilde{x},\tilde{y})\in P}{\mathrm{median}}\left(\frac{I_{c}\left(\tilde{x},\tilde{y}\right)}{I_{\mathrm{avg}}\left(\tilde{x},\tilde{y}\right)}\right)\;.$$

In order to robustly calculate the ratio $I_{c}/I_{\mathrm{avg}}$, the median of the ratio is used.

### 3.8. Update Rule

After the initial values for the normal n and albedo A are calculated, as shown in Section 3.6 and Section 3.7, the calculations are iterated several times. First, the surface normal is calculated according to the procedure shown in Section 3.3. The calculated surface normal is denoted as $n^{\prime }$, and the surface normal of the previous step is denoted as $n^{\prime \prime }$. Instead of using $n^{\prime }$, the new surface normal n for the next step is calculated as Equation (27).

(27) $$n=\left(1-\alpha_{n}\right)n^{\prime }+\alpha_{n}n^{\prime \prime }\;.$$

The constant $\alpha_{n}$ stabilizes the update of the surface normal, resulting in robust optimization. Actually, instead of Equation (27), we implemented our software as follows.

(28) $$\left(\begin{array}{c} n_{x}\\ n_{y}\\ n_{z} \end{array}\right)={\left(\begin{array}{ccc} rl_{x0} & rl_{y0} & rl_{z0}\\ \vdots & \vdots & \vdots \\ rl_{x,c-1} & rl_{y,c-1} & rl_{z,c-1}\\ drl_{x,c} & drl_{y,c} & drl_{z,c}\\ rl_{x,c+1} & rl_{y,c+1} & rl_{z,c+1}\\ \vdots & \vdots & \vdots \\ rl_{x6} & rl_{y6} & rl_{z6}\\ {\tilde{\alpha }}_{n} & 0 & 0\\ 0 & {\tilde{\alpha }}_{n} & 0\\ 0 & 0 & {\tilde{\alpha }}_{n} \end{array}\right)}^{+}\left(\begin{array}{c} rI_{0}\left(x,y\right)/\left(A_{0}\left(x,y\right)+\varepsilon_{1}\right)\\ \vdots \\ rI_{c-1}\left(x,y\right)/\left(A_{c-1}\left(x,y\right)+\varepsilon_{1}\right)\\ drI_{c}\left(x,y\right)/\left(A_{c}\left(x,y\right)+\varepsilon_{1}\right)\\ rI_{c+1}\left(x,y\right)/\left(A_{c+1}\left(x,y\right)+\varepsilon_{1}\right)\\ \vdots \\ rI_{6}\left(x,y\right)/\left(A_{6}\left(x,y\right)+\varepsilon_{1}\right)\\ {\tilde{\alpha }}_{n}{\tilde{n}}_{x}\\ {\tilde{\alpha }}_{n}{\tilde{n}}_{y}\\ {\tilde{\alpha }}_{n}{\tilde{n}}_{z} \end{array}\right)\;.$$

Here, the surface normal of the previous iteration is represented as $\left({\tilde{n}}_{x},{\tilde{n}}_{y},{\tilde{n}}_{z}\right)$ and the updated surface normal to be taken over to the next iteration is represented as $\left(n_{x},n_{y},n_{z}\right)$. After solving this equation, the obtained surface normal is normalized.

Here, we have employed additional weight *r*. This weight depends on the number of valid channels for each pixel. If there are no shadows and speculars in all seven channels, we set *r* as a large number, so that the surface normal calculated by photometric stereo equation becomes much more important than the surface normal of the previous iteration $\left({\tilde{n}}_{x},{\tilde{n}}_{y},{\tilde{n}}_{z}\right)$. If there are many invalid channels, the surface normal calculated by photometric stereo equation becomes unreliable, thus we set *r* small so that surface normal will be unchanged. We define *r* as follows using the parameter *w*.

(29) $$r={\left(\frac{\max(v-2,0)}{7-2}\right)}^{w}\;.$$

Here, *v* is the number of valid channels. We found empirically that w>1 is good for stable computation.

Next, albedo is calculated according to the procedure shown in Section 3.3. The calculated albedo is denoted as $A^{\prime }$, and the albedo of the previous step is denoted as $A^{\prime \prime }$. The update rule for albedo is shown in Equation (30).

(30) $$A=\left(1-\alpha_{a}\right)A^{\prime }+\alpha_{a}A^{\prime \prime }\;.$$

The constant value $\alpha_{a}$ stabilizes the optimization.

Instead of using Equation (30), we implemented this process as follows.

(31) $$A_{c}=\frac{\left(1-{\tilde{\alpha }}_{a}\right)I_{c}+{\tilde{\alpha }}_{a}{\tilde{A}}_{c}}{\left(1-{\tilde{\alpha }}_{a}\right)\left(l_{c}^{⊤}n\right)+{\tilde{\alpha }}_{a}}\;.$$

This is a weighted sum of Equation (17) and the previously calculated albedo ${\tilde{A}}_{c}$ with the weight ${\tilde{\alpha }}_{a}$. Note that Equation (31) is calculated if channel *c* is marked as valid through the process shown in Section 3.9, and $A_{c}={\tilde{A}}_{c}$ is used if it is invalid.

### 3.9. Outlier Detection

Detecting specular reflection in color photometric stereo problems is difficult. One of the common approaches for detecting specular reflection is to use color. The colors of diffuse reflection and specular reflection are usually different; thus, the diffuse reflection and specular reflection can be separated when the scene is illuminated by a nearly white light source. However, the color photometric stereo illuminates the object with three different colors, and thus, the color-based approach cannot solve the problem. Another approach is to use principal component analysis or singular value decomposition, which represents the image with three orthonormal bases. However, the color of each light is different in color photometric stereo approach, and thus, the images cannot be represented by a linear sum of three bases. As a result, the remaining approach is to use the strong brightness change caused at specular reflection.

Therefore, we have no choice but to use thresholding approach for outlier (specular / shadow) detection. Suppose that the maximum brightness of the object for all channels is $I_{\max}$ and the minimum is $I_{\min}$. We use $T_{\max}=I_{\max}-t_{\max}$ and $T_{\min}=I_{\min}+t_{\min}$ as thresholds, where $t_{\max}$ and $t_{\min}$ are small positive constants. Outlier map *N*, which is 1 for outlier and 0 for valid pixel, is designed as follows.

(32) $${\tilde{N}}_{c}\left(x,y\right)=\left\{\begin{array}{cc} 1 & \mathrm{if}\;I_{c}\left(x,y\right)>T_{\max}\;\mathrm{or}\;I_{c}\left(x,y\right)<T_{\min}\;,\\ 0 & \mathrm{otherwise}\;, \end{array}\right.$$

(33) $$N_{c}=\mathrm{dilation}\left({\tilde{N}}_{c}\right)\;.$$

Here, “dilation” is an operator which dilates the “1” pixels, which is a well-known operator in binary image processing, which we skip to explain.

### 3.10. Calculating Height from Surface Normal

In this section, we briefly introduce the procedure to calculate the height from surface normal. Here, we assume orthographic projection, and the perspective projection case is shown in the literature [27]. More details are given in the literature [27,28,29].

The shape is represented as the height *H* set for each pixel. The partial derivatives of the heights with respect to *x* and *y* are called gradient, and represented as *p* and *q*, respectively.

(34) $$p=H_{x}=\frac{\partial H}{\partial x},\;q=H_{y}=\frac{\partial H}{\partial y}\;.$$

The surface normal n is represented by these gradients as shown below.

(35) $$n=\frac{{\left(-p,-q,1\right)}^{⊤}}{\sqrt{p^{2}+q^{2}+1}}\;.$$

The cost function that relates the surface normal to the height is shown below.

(36) $$\int \;\;\int {\left(H_{x}-p\right)}^{2}+{\left(H_{y}-q\right)}^{2}dxdy\;.$$

The Euler equation (Euler-Lagrange differential equation) that minimizes the equation

(37) $$\int \;\;\int F(u,u_{x},u_{y})dxdy\;,$$

can be expressed as

(38) $$F_{u}-\frac{\partial F_{u_{x}}}{\partial x}-\frac{\partial F_{u_{y}}}{\partial y}=0\;.$$

As for *H*, the Euler equation that minimizes Equation (36) is derived as follows:

(39) $$H_{xx}+H_{yy}-p_{x}-q_{y}=0\;.$$

Here, $H_{xx}$ and $H_{yy}$ can be discretized as follows:

(40) $$\begin{array}{ccc} H_{xx} & = & H(x+1,y)+H(x-1,y)-2H(x,y) \end{array}$$

(41) $$\begin{array}{ccc} H_{yy} & = & H(x,y+1)+H(x,y-1)-2H(x,y)\;. \end{array}$$

Thus, substituting Equations (40) and (41) into Equation (39) yields the following equation.

(42) $$H\left(x,y\right)=\frac{1}{4}\left(H\left(x+1,y\right)+H\left(x-1,y\right)+H\left(x,y+1\right)+H\left(x,y-1\right)\right)-\frac{1}{4}\left(p_{x}\left(x,y\right)+q_{y}\left(x,y\right)\right)\;.$$

As is shown in Equation (35), the gradients *p* and *q* are calculated from the surface normal n. The partial differentiation of gradients used for Equation (42) is discretized as follows.

(43) $$\begin{array}{ccc} p_{x}\left(x,y\right) & = & p(x+1,y)-p(x-1,y)\;,\\ q_{y}\left(x,y\right) & = & q(x,y+1)-q(x,y-1)\;. \end{array}$$

After computing Equation (43), we solve Equation (42) to determine the height *H*. In this paper, we solve Equation (42) using the successive over-relaxation method, but any other methods are also applicable, such as Fourier transform [30] or preconditioned conjugate gradient [31].

### 3.11. Channel Crosstalk

In an instrument that independently uses signals of two or more channels, signal leaking from one channel to another is called crosstalk. Our experiment uses a multi-band camera that has seven channels and detects undesired colors of other channels. The undesired effect of a color camera is called channel crosstalk [11,32,33,34].

**Figure 5:**
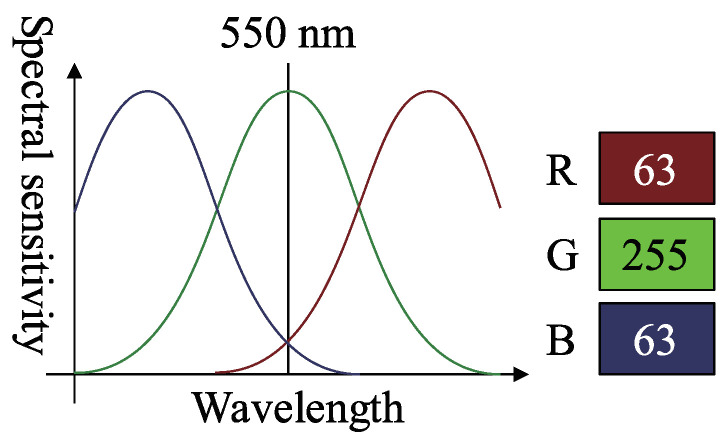
Example of camera spectral sensitivity which has channel crosstalk.

**Figure 6:**
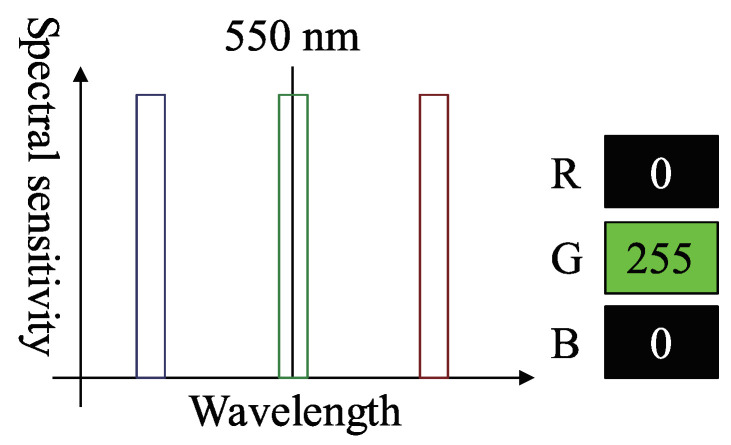
Example of camera spectral sensitivity which does not have channel crosstalk.

Figure 5 is an example of a three-band RGB camera that detects 550 nm green light as (R,G,B)=(63,255,63). This signal should be (R,G,B)=(0,255,0) since the observed green light wavelength is 550 nm. As shown in Figure 5, the bandwidth of each spectral sensitivity is wide, and thus, has some overlaps; therefore, the R and B channels also detect the color of green light. Color photometric stereo assumes that the sensor has no channel crosstalk, as shown in Figure 6; thus, we must remove channel crosstalk.

To detect the channel crosstalk, we use a diffuse white reflectance standard, which has flat spectral reflectance for each wavelength. The seven-band camera captures seven images of the diffuse white reflectance standard illuminated by one of the seven light sources, which are lit one-by-one. A single channel is sensitive to each light; thus, the signals of other channels are caused by the crosstalk.

Channel crosstalk can be represented by a color mixing matrix X. Since we use a seven-band camera, the size of matrix X is 7×7. Let us denote the ideal signal without channel crosstalk as $d_{i}$. This seven-dimensional column vector $d_{i}$ becomes $d_{o}$ because it is affected by channel crosstalk. The relation between these signals and the color mixing matrix is as follows.

(44) $$d_{o}=Xd_{i}\;.$$

The original signal $d_{i}$ can be recovered from the captured signal $d_{o}$ as follows.

(45) $$d_{i}=X^{-1}d_{o}\;.$$

The color mixing matrix X should be obtained prior to the measurement, and the input image should be converted by the inverse of the color mixing matrix $X^{-1}$ before applying the proposed algorithm.

Suppose that we look at the 0th channel of the diffuse white reflectance standard illuminated by the 0th light with narrow-band wavelength. Ideally, the signal should be zero for each channel, except the 0th channel. We define the value of the 0th channel as 1. Namely, the ideal signal $d_{i}={\left(1,0,0,0,0,0,0\right)}^{T}$ becomes $d_{o}={\left(w_{0,0},w_{1,0},\cdots ,w_{6,0}\right)}^{T}$ after observation.

(46) $${\left(\begin{array}{ccccc} w_{0,0} & w_{1,0} & w_{2,0} & \cdots & w_{6,0} \end{array}\right)}^{⊤}=X{\left(\begin{array}{ccccc} 1 & 0 & 0 & \cdots & 0 \end{array}\right)}^{⊤}\;.$$

Similarly, the diffuse white reflectance standard illuminated by the 1st light is expressed as follows.

(47) $${\left(\begin{array}{ccccc} w_{0,1} & w_{1,1} & w_{2,1} & \cdots & w_{6,1} \end{array}\right)}^{⊤}=X{\left(\begin{array}{ccccc} 0 & 1 & 0 & \cdots & 0 \end{array}\right)}^{⊤}\;.$$

This procedure is repeated until the 6th light. The following equation expresses the whole measurement, which is conducted seven times.

(48) $$\left(\begin{array}{cccc} w_{0,0} & w_{0,1} & \ldots & w_{0,6}\\ w_{1,0} & w_{1,1} & \ldots & w_{1,6}\\ \vdots & \vdots & \ddots & \vdots \\ w_{6,0} & w_{6,1} & \ldots & w_{6,6} \end{array}\right)=X\left(\begin{array}{cccc} 1 & 0 & \ldots & 0\\ 0 & 1 & \ldots & 0\\ \vdots & \vdots & \ddots & \vdots \\ 0 & 0 & \ldots & 1 \end{array}\right)\;.$$

As a result, the color mixing matrix X is obtained as follows.

(49) $$X=\left(\begin{array}{cccc} w_{0,0} & w_{0,1} & \ldots & w_{0,6}\\ w_{1,0} & w_{1,1} & \ldots & w_{1,6}\\ \vdots & \vdots & \ddots & \vdots \\ w_{6,0} & w_{6,1} & \ldots & w_{6,6} \end{array}\right)\;.$$

The inverse of the color mixing matrix $X^{-1}$ can cancel the channel crosstalk of the observed signal. The output ideal signal $d_{i}$ is calibrated such that the signal of the diffuse white reflectance standard would be (1,1,⋯,1).

## 4. Experiment

### 4.1. Experimental Setup

**Figure 7:**
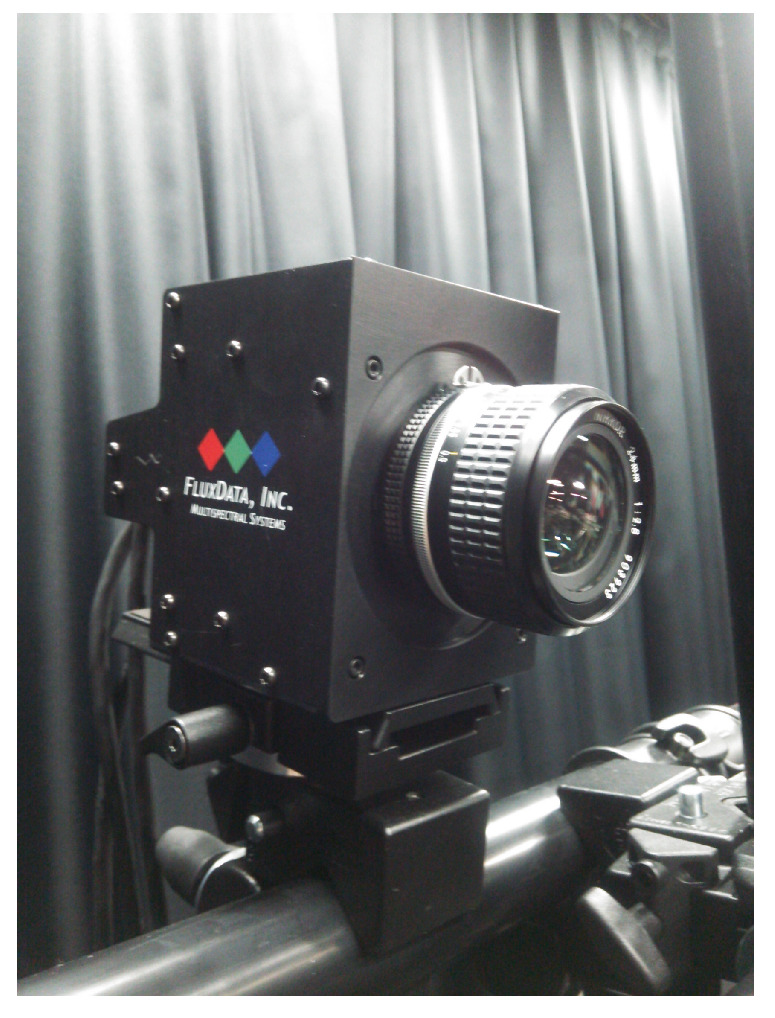
Multispectral camera “FluxData FD-1665 (USA)”.

**Figure 8:**
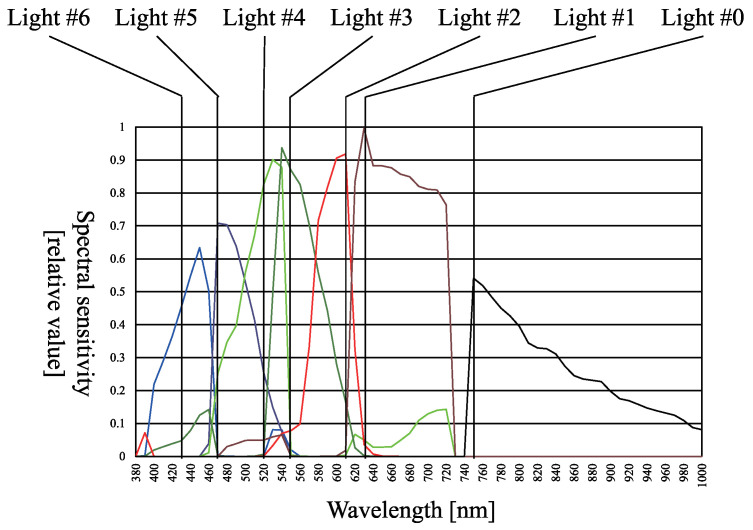
Spectral sensitivity of multispectral camera and peak wavelength of each light sources.

**Figure 9:**
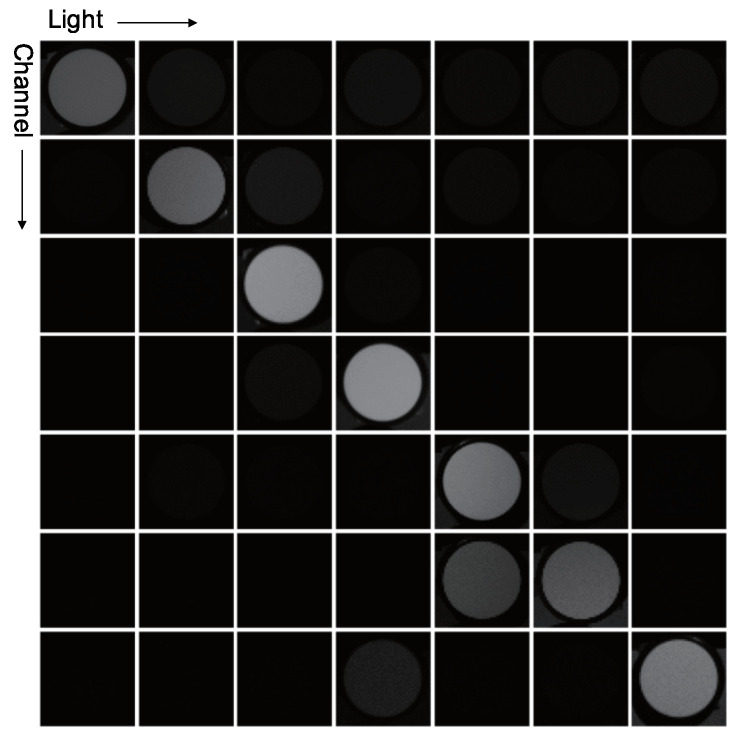
The obtained color mixing matrix for canceling channel crosstalk. The average brightness of white reflectance stardard becomes the color mixing matrix. The matrix will be diagonal matrix if there are no channel crosstalk, however non-diagonal element is slightly bright due to the channel crosstalk.

The camera used for this experiment is an FD-1665 3CCD multi-spectral camera by FluxData, Inc., USA, as shown in Figure 7. It comprises two color sensors and a near-infrared (NIR) sensor. Each sensor is sensitive to its respective wavelength, i.e., each color sensor can record the components from three channels, and the NIR sensor can record the components from one channel. Figure 8 shows the spectral sensitivity of the camera. As shown in Figure 8, channel crosstalk occurred among all camera channels. Therefore, the method shown in Section 3.11 is used to remove the channel crosstalk in the photographed input image. The diffuse white reflectance standard is used to obtain the color mixing matrix shown in Figure 9, where the row denotes the channel number and the column denotes the light number. The color mixing matrix is created using the average value of the diffuse white reflectance standard.

Table 1 shows the full width at half maximum (FWHM) for each light source used in this experiment.

The light source directions were determined prior to the experiment by photographing a mirrored ball. The locations of the light sources and the camera were then left unchanged.

**Figure 10:**
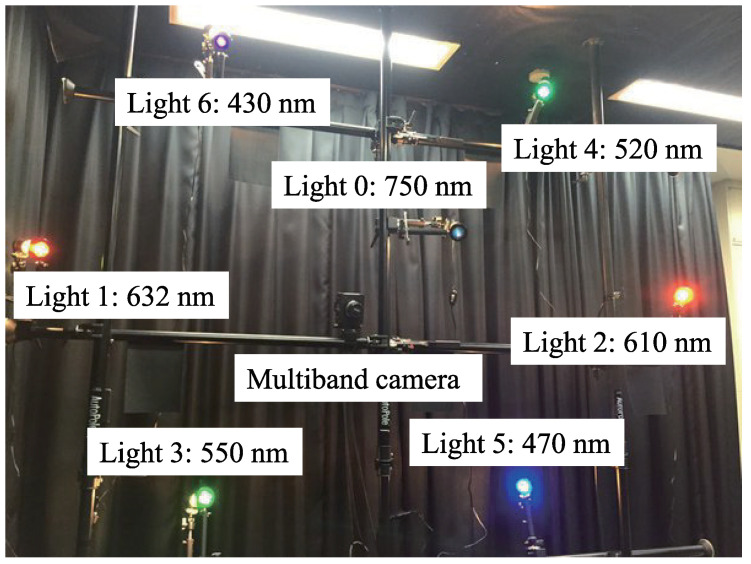
Experimental setup with 7 light sources with different wavelengths and a single 7-band multispectral camera.

The experiment was conducted in a darkroom. To increase the amount of supplementary information obtained for objects with narrow-wavelength regions, light sources of close wavelength were positioned opposite to each other. The NIR light source was placed next to the camera. Figure 10 shows a diagram of the experiment.

**Figure 11:**
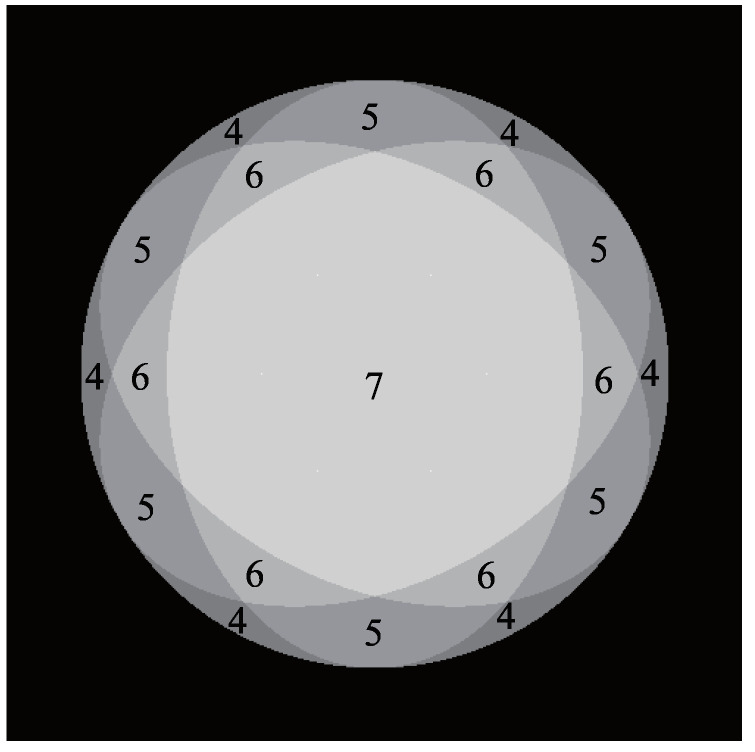
Gaussian sphere representation of the surface normal where the north pole is the center of this picture. The number indicates how many light sources are lit for each direction of surface normal.

Each point on the object’s surface must always be illuminated by more than three light sources for the photometric stereo method. If there are six light sources, any point on the surface can be illuminated by at least three light sources [35]. Additionally, when specular reflection occurs, one picture that can be used for the photometric stereo method is eliminated. Therefore, the NIR light source is placed next to the camera so that each point is illuminated by at least four light sources. Figure 11 is Gaussian sphere representation of the surface normal, where the number of each region represents the number of light sources illuminated.

**Figure 12:**
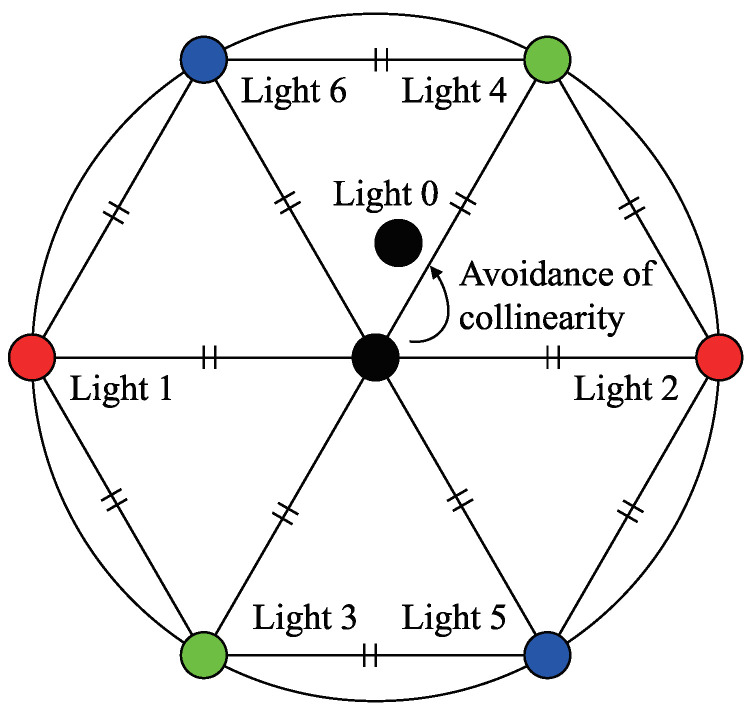
Schematic illustration of the geometrical location of seven light sources. Six lights are placed at each apex of a regular hexagon. Multispectral camera is placed at the center of the hexagon. Infrared light is placed near the camera.

In the photometric stereo method, precision increases when the angle between the light sources is widened, i.e., the baseline is lengthened, because it increases the shading contrast. However, when the baseline is lengthened, the shadow area increases. The locations of the light sources must, therefore, be limited to a certain solid angle. When seven points are placed within a fixed circle, the placement of the points must be as far from each other as possible to comprise the vertices of a regular hexagon and its center, as shown in Figure 12. Therefore, when placing seven light sources within a limited area for the photometric stereo method, it is optimal to place them at the vertices of a regular hexagon and its center.

However, when three of the light sources selected from these seven lights are placed on the same straight line, or more precisely, when the three light source vectors are coplanar, the surface normal cannot be estimated by combining the three light sources. This is because combining these three light sources causes the light source matrix to degenerate. Suppose that the surface normal n is illuminated by light sources $l_{0}$, $l_{1}$, and $l_{2}$, and is observed as the pixel brightnesses $I_{0}$, $I_{1}$, and $I_{2}$, respectively, while ignoring the shadow. If the light source directions are known, the surface normal can be obtained from following equation if there is an inverse of 3×3 light source matrix ${\left(l_{0},l_{1},l_{2}\right)}^{⊤}$.

(50) $$\left(\begin{array}{c} l_{0}^{⊤}\\ l_{1}^{⊤}\\ l_{2}^{⊤} \end{array}\right)(n)=\left(\begin{array}{c} I_{0}\\ I_{1}\\ I_{2} \end{array}\right)$$

The determinant of ${\left(l_{0},l_{1},l_{2}\right)}^{⊤}$ is the scalar triple product $l_{0}\cdot \left(l_{1}\times l_{2}\right)$. If $l_{0}$, $l_{1}$, and $l_{2}$ are coplanar, the vector $l_{1}\times l_{2}$ becomes orthogonal to the vector $l_{0}$, thus the determinant becomes zero. Although two-light photometric stereo exists [36], it is better to avoid three lights to be coplanar if we have more than two lights. Therefore, the NIR light source is placed at a small distance from the center of the regular hexagon so that no three light sources are on the same straight line. The camera is placed at the center of the regular hexagon.

### 4.2. Experimental Result

The computation time of the main part of the algorithm (i.e., excluding the computation time of calculating the initial value) is about ten seconds for ordinary object and ordinary computer with single thread and without any fine tuning to the source code.

As for all experimental results shown in this section, we used ${\tilde{\alpha }}_{n}=0.1$ and ${\tilde{\alpha }}_{a}=0.99$. These two parameters are the most important parameters which affect the final result, and other parameters are relatively less influential in comparison to these parameters. We used 4 for the standard deviation of Gaussian filter for smoothing the surface normal, and 15×15 and 11×11 for the window size of median filter of surface normal and albedo, respectively. The iteration number was set to be 2. We used w=16, d=0.0001, $\epsilon_{1}=0.001$, and $\epsilon_{2}=0.1$. The abovementioned parameters are the all parameters used in the main process.

As for calculating the initial albedo, we used 2 for the standard deviation of spatial parameter, and 20 for the standard deviation of intensity parameter for the bilateral filter. When applying the bilateral filter, the pixel brightness of outlier is scaled by 0.1 when calculating the weighted sum. The iteration number of the bilateral filter is set to be 10. As for calculating the scale, in order to avoid division-by-zero error, $I_{\mathrm{avg}}\leq 0.1$ is not used for calculating Equation (26). As for calculating the initial normal, smoothing filter is applied twice: First it is applied to the height data and next it is applied to the surface normal. As for smoothing, 3×3 box filter is used, and the iteration number was set to be 100, for both the height and the normal. As for outlier detection, $t_{\max}=15$ and $t_{\min}=5$ are used. The number of dilation is set to be 1. The abovementioned parameters are the all parameters used in calculating the initial values.

**Figure 13:**
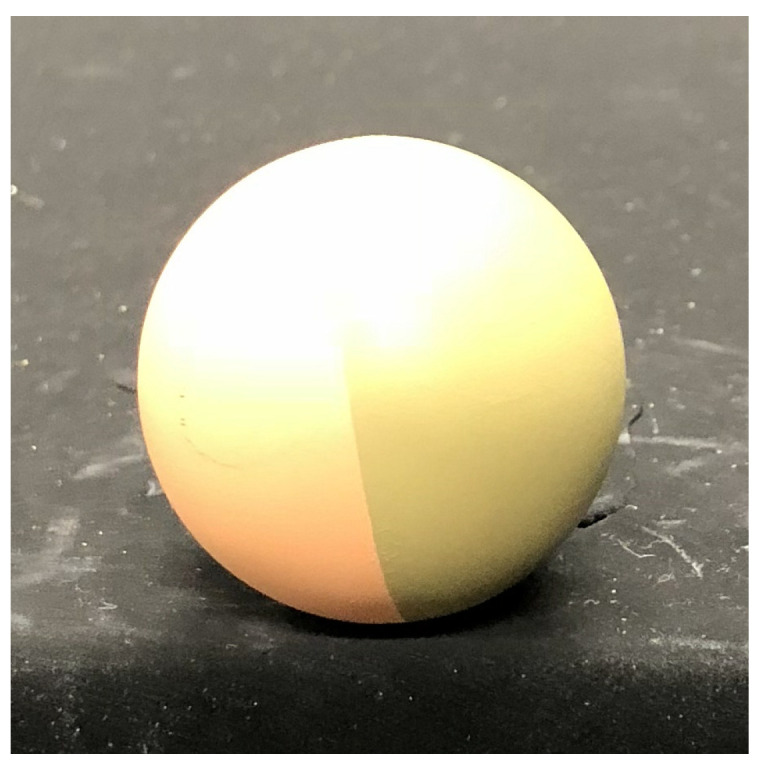
A spherical object with two different colors is used for evaluation since we know the mathematically true surface normal of the sphere.

**Figure 14:**
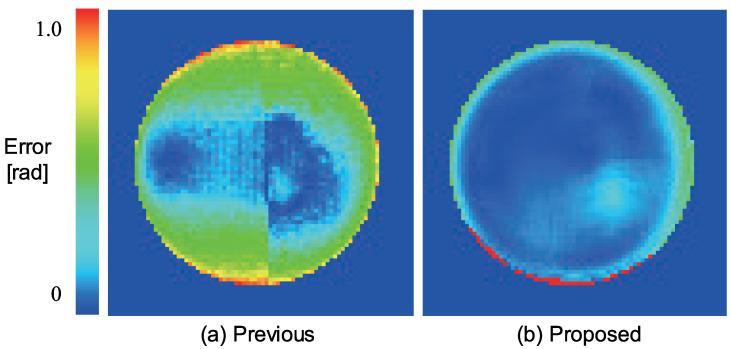
The error map of the sphere where the error is represented as angular difference between estimated value and ground truth (red: large, blue: small): (**a**) Naive color photometric stereo and (**b**) our method.

First, we measured a plastic sphere to evaluate our system. The spherical object shown in Figure 13 consists of two types of albedos. Figure 14 shows the error map with pseudo-color representation. The error is evaluated as an angle between the estimated surface normal and the true surface normal. We measured a sphere because its true surface normal can be obtained from the mathematical expression of the sphere. We compared our method with the so-called “naive color photometric stereo.” In this paper, we define the color photometric stereo that assumes white objects as target as naive color photometric stereo. The generalized color photometric stereo problem shown in Equation (6) has nine unknown parameters; however, naive color photometric stereo has three unknown parameters: single albedo value (one parameter) and 3D surface normal (two parameters since it is constrained to be a unit vector). Therefore, naive color photometric stereo directly solves the linear equation even if the image is captured by a three-band color camera. Naive color photometric stereo robustly estimates the surface normal of white shirts, white dresses, and so on. The mean error of naive color photometric stereo (Figure 14a) were 0.343 [rad]. Our method overwhelms the previous approach, and our mean error (Figure 14b) was 0.148 [rad].

**Figure 15:**
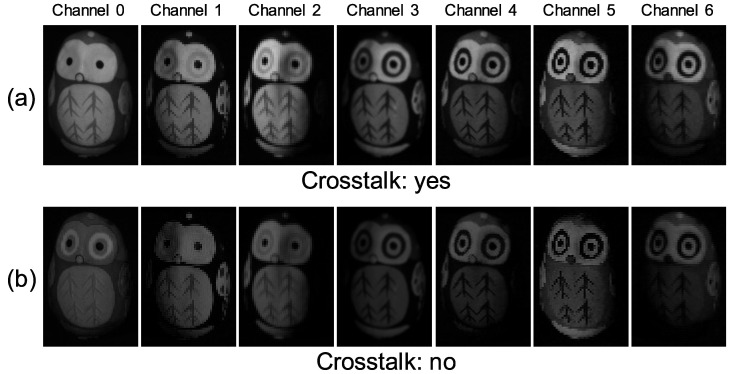
Obtained multi-band image [owl]: (**a**) Captured image and (**b**) image after cancelling channel crosstalk. If you look carefully, you may notice that the channel crosstalk is removed. However, the difference is difficult to recognize since the crosstalk is small as is shown in Figure 9.

**Figure 16:**
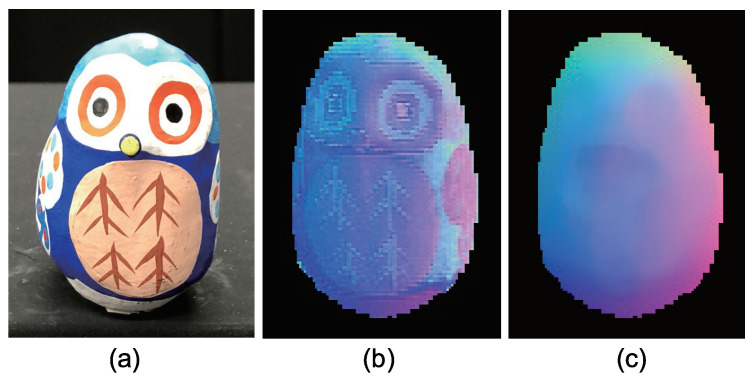
The results for owl object, which only causes diffuse reflection. Estimated surface normal [owl]: (**a**) Target object, (**b**) surface normal of naive color photometric stereo, and (**c**) surface normal of our method. The proposed method is not affected by the albedo difference.

**Figure 17:**
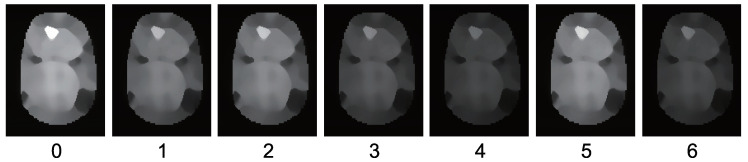
The results for owl object, which only causes diffuse reflection. Estimated albedo is shown, which is smooth enough.

**Figure 18:**
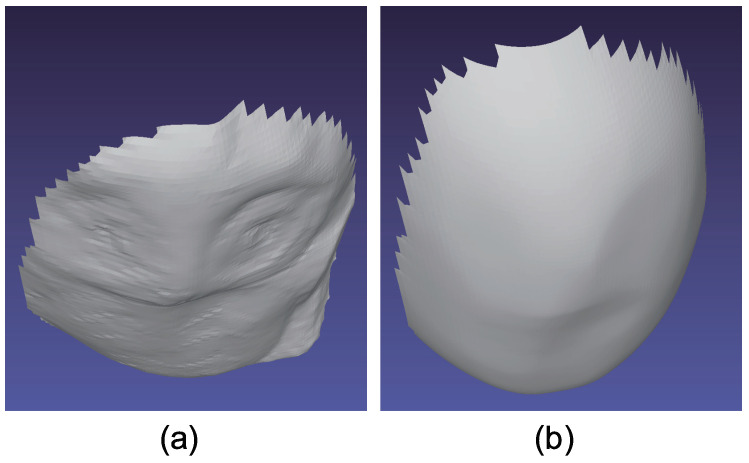
The results for owl object, which only causes diffuse reflection. Estimated geometry [owl]: (**a**) Naive color photometric stereo and (**b**) our method. The proposed method is not affected by the albedo difference.

Figure 15 shows the seven-channel image of an owl figurine (Figure 16a). The captured image shown in Figure 15a is contaminated by channel crosstalk, and thus, we cancelled it, which resulted in Figure 15b. The surface normal estimated by naive color photometric stereo is shown in Figure 16b and that estimated by our method is shown in Figure 16c. As usual, the *x*, *y*, and *z* axes of the surface normal are linearly converted to R, G, and B for the pseudo-color representation of the surface normal. The estimated albedo is shown in Figure 17. The shapes obtained by naive color photometric stereo and by our method are shown in Figure 18a,b, respectively.

**Figure 19:**
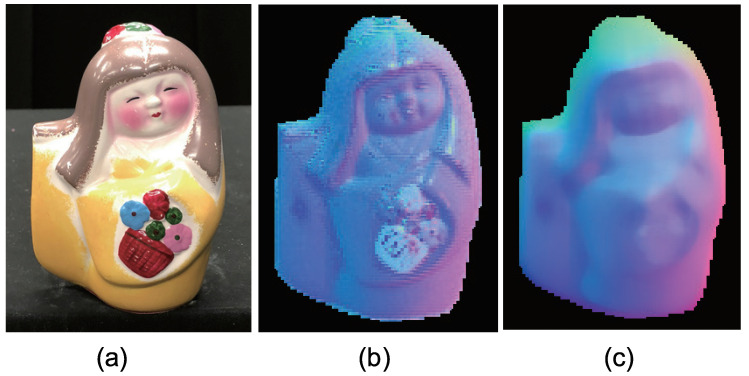
The results for doll object, which causes strong specular reflection. Estimated surface normal [doll]: (**a**) Target object, (**b**) surface normal of naive color photometric stereo, and (**c**) surface normal of our method. The proposed method is not affected by the albedo difference appears at the flower basket.

**Figure 20:**
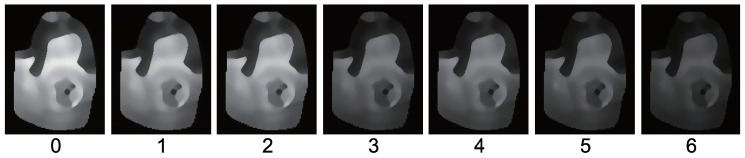
The results for doll object, which causes strong specular reflection. Estimated albedo is shown, which is smooth enough.

**Figure 21:**
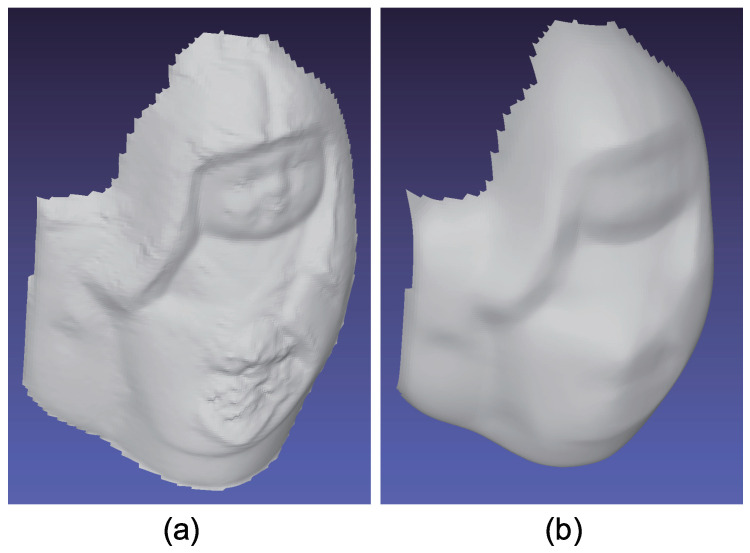
The results for doll object, which causes strong specular reflection. Estimated geometry [doll]: (**a**) Naive color photometric stereo and (**b**) our method. The proposed method is not affected by the albedo difference appears at the flower basket.

**Figure 22:**
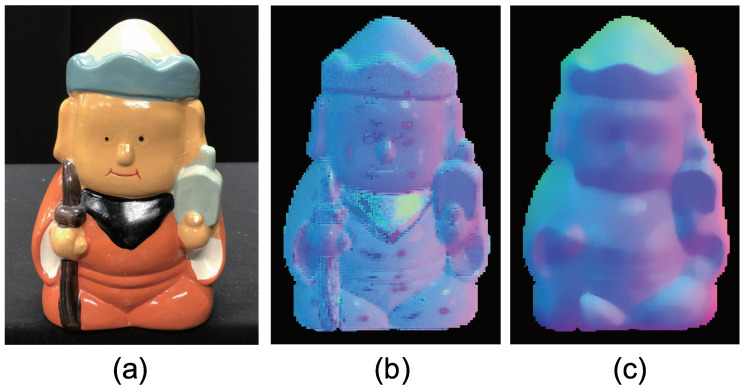
The results for buddha object, which causes strong specular reflection. Estimated surface normal [Buddha]: (**a**) Target object, (**b**) surface normal of naive color photometric stereo, and (**c**) surface normal of our method. The proposed method can smooth the surface normal of the scarf whose surface normal is unreliable due to black paint.

**Figure 23:**
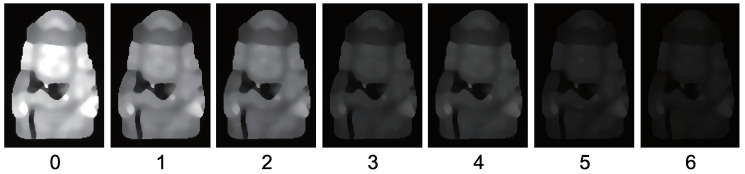
The results for buddha object, which only causes strong specular reflection. Estimated albedo is shown, which is smooth enough.

**Figure 24:**
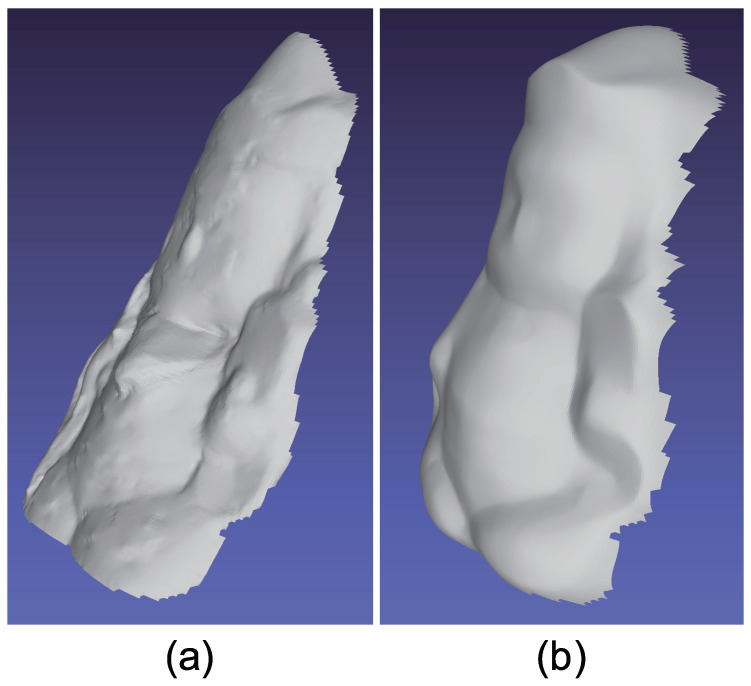
The results for buddha object, which causes strong specular reflection. Estimated geometry [Buddha]: (**a**) Naive color photometric stereo and (**b**) our method. The proposed method can smooth the surface normal of the scarf whose surface normal is unreliable due to black paint.

The same experiment was also conducted with another multicolored object. The results with the doll and Buddha figurines are shown in Figure 19, Figure 20, Figure 21, Figure 22, Figure 23 and Figure 24, respectively.

**Figure 25:**
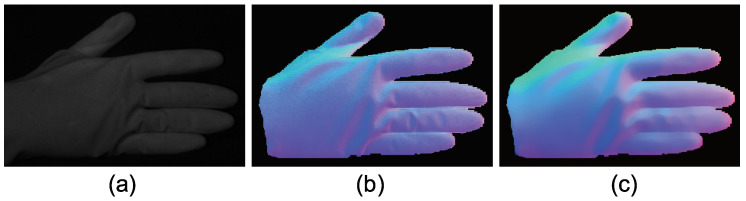
The results for hand with glove. Estimated surface normal [pose 1]: (**a**) One of the seven channel images, (**b**) estimated surface normal [naive color photometric stereo], and (**c**) estimated surface normal [our method]. Both color photometric stereos can estimate the surface normal of the dynamically deforming object.

**Figure 26:**
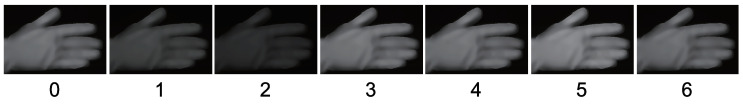
The results for hand with glove. Estimated albedo [pose 1] is shown, which is smooth enough.

**Figure 27:**
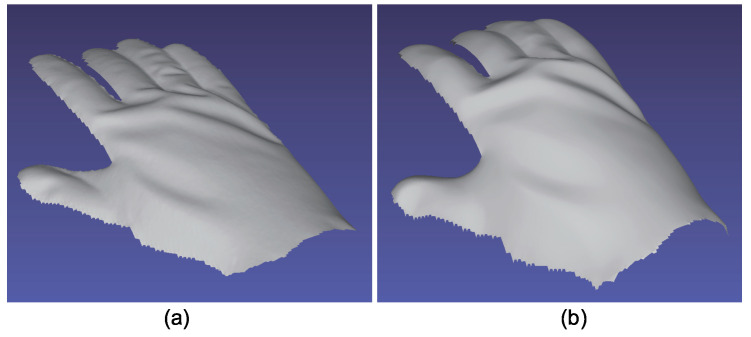
The results for hand with glove. Estimated geometry [pose 1]: (**a**) Estimated geometry [naive color photometric stereo] and (**b**) estimated geometry [our method]. Both color photometric stereos can estimate the surface normal of the dynamically deforming object.

**Figure 28:**
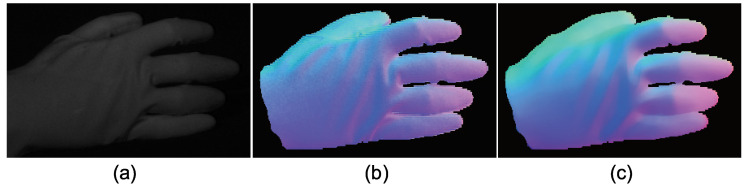
The results for hand with glove. Estimated surface normal [pose 2]: (**a**) One of the seven channel images, (**b**) estimated surface normal [naive color photometric stereo], and (**c**) estimated surface normal [our method]. Both color photometric stereos can estimate the surface normal of the dynamically deforming object.

**Figure 29:**
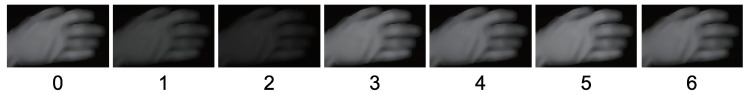
The results for hand with glove. Estimated albedo [pose 2] is shown, which is smooth enough.

**Figure 30:**
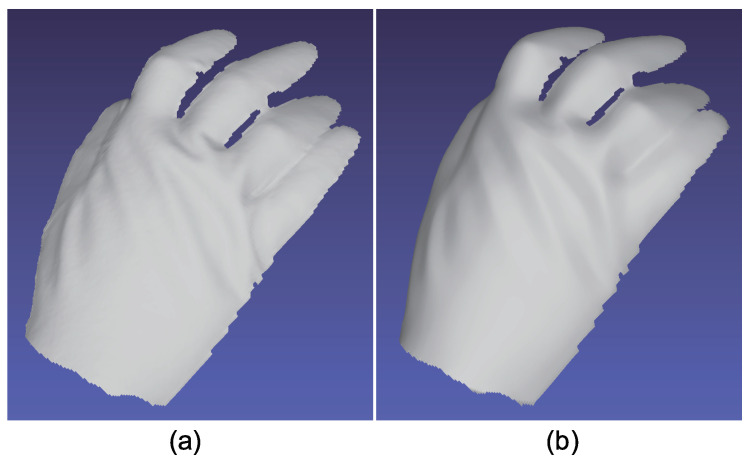
The results for hand with glove. Estimated geometry [pose 2]: (**a**) Estimated geometry [naive color photometric stereo] and (**b**) estimated geometry [our method]. Both color photometric stereos can estimate the surface normal of the dynamically deforming object.

The advantage of color photometric stereo is that the surface normal of dynamic objects can be obtained. Most existing color photometric stereo methods measure white shirts, white dresses, etc., to verify that these methods can be applied to dynamically deforming objects. Due to the small size of the darkroom, we measured a glove instead of clothes. Figure 25, Figure 26 and Figure 27 show the measurement results, and Figure 28, Figure 29 and Figure 30 show the results of the same object but differently deformed.

### 4.3. Discussion

**Figure 31:**
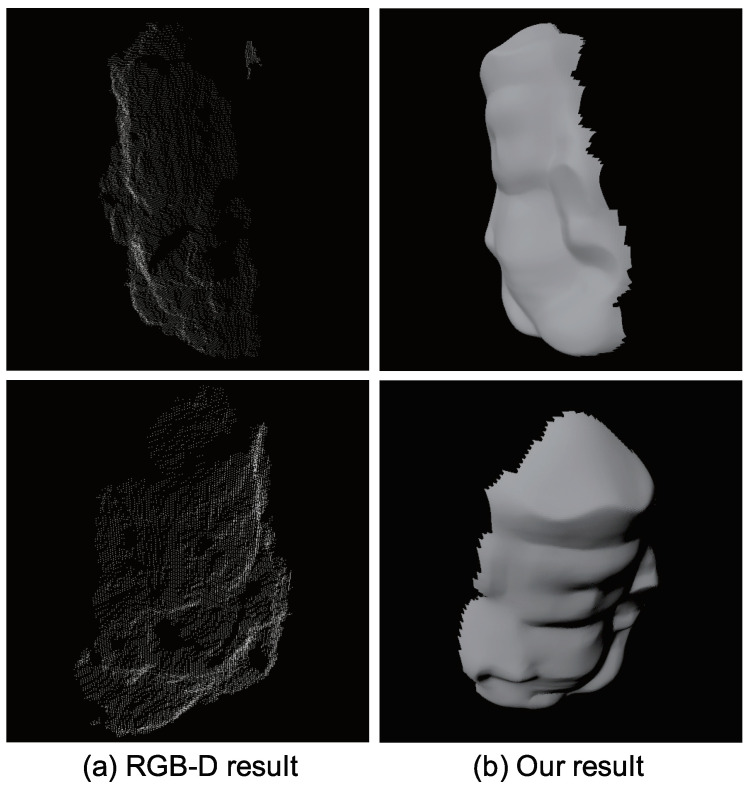
Comparison with off-the-shelf depth sensor: (**a**) Result of off-the-shelf depth sensor and (**b**) result of our method. The depth sensor can estimate the 3D coordinate of vertices successfully and the photometric stereo can estimate the surface normal successfully.

Figure 31a shows the result of Microsoft Kinect sensor. For comparison, our result is shown in Figure 31b. Kinect measures the depth and photometric stereo measures the surface normal. These two sensors are fundamentally different, however, since Kinect is a well-known commercial product of shape measurement, we think beneficial to show Figure 31 for the readers.

**Figure 32:**
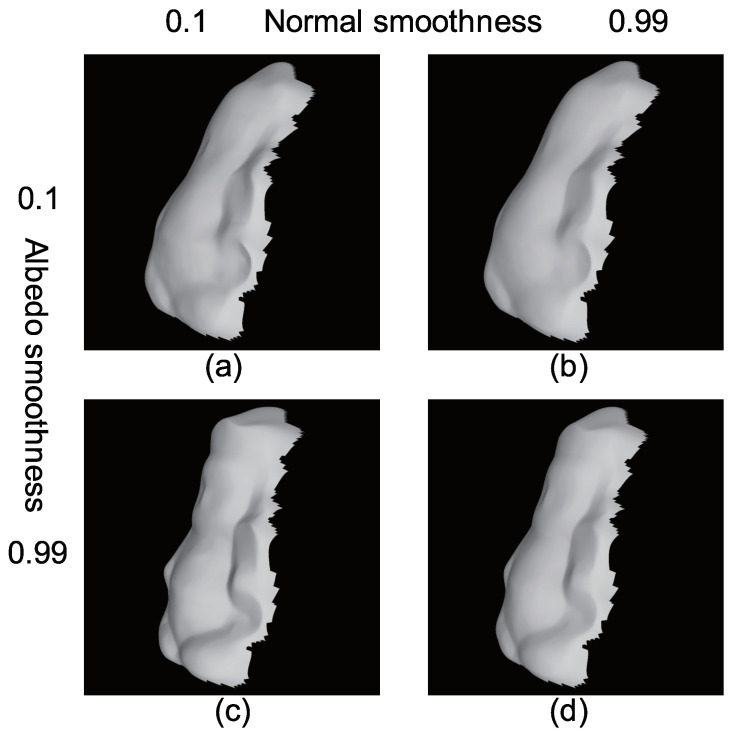
How the weight of smoothness term affects the results: (**a**) Sharp normal and sharp albedo, (**b**) smooth normal and sharp albedo, (**c**) sharp normal and smooth albedo, and (**d**) smooth normal and smooth albedo.

Figure 32 shows how the surface normal is affected by the parameters (Equations (28) and (31)). Figure 32a,b are the results when ${\tilde{\alpha }}_{a}=0.1$, while Figure 32c,d are the results when ${\tilde{\alpha }}_{a}=0.99$. Figure 32a,c are the results when ${\tilde{\alpha }}_{n}=0.1$, while Figure 32b,d are the results when ${\tilde{\alpha }}_{n}=0.99$. Figure 32b is smoother than Figure 32a, and Figure 32d is smoother than Figure 32c, since the smoothness constraint of surface normal is stronger. Figure 32a is smoother than Figure 32c, and Figure 32b is smoother than Figure 32d, since the albedo is not smooth, which means that the surface normal becomes relatively smooth. Although Figure 17, Figure 20, Figure 23, Figure 26, and Figure 29 show over-smoothed result of albedo, it is an adequate way to smooth the albedo in order to obtain sharp features of surface normal.

**Figure 33:**
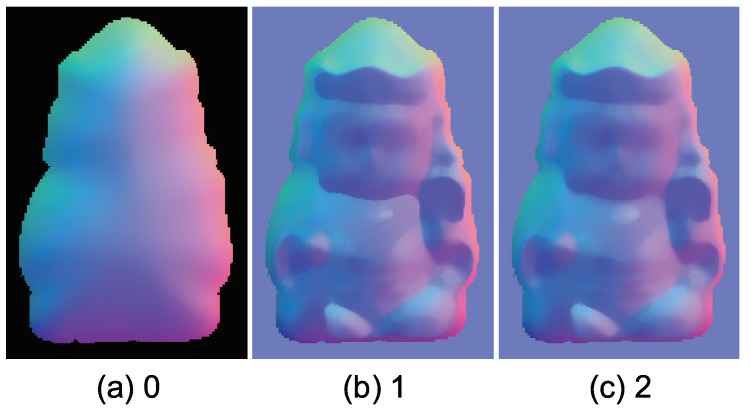
Intermediate state of surface normal through the proposed method: (**a**) Initial value of the surface normal, (**b**) the surface normal after 1 iteration, and (**c**) surface normal after 2 iterations. The proposed method is stable and coverges fast.

Figure 33a shows the initial value of the surface normal, and Figure 33b,c shows how the surface normal is updated. This figure proves that our algorithm is stable since it converges quickly.

As shown in Figure 14, our method is robust to multiple types of albedos. On the other hand, as shown in Figure 16, Figure 17, Figure 18, Figure 19, Figure 20, Figure 21, Figure 22, Figure 23, Figure 24, Figure 25, Figure 26, Figure 27, Figure 28, Figure 29 and Figure 30, our method over-smoothens the detailed surface structure. The generalized color photometric stereo problem shown in Equation (6) has nine unknown parameters; however, naive color photometric stereo has three unknown parameters, as stated in Section 4.2. Naive color photometric stereo robustly estimates the surface normal of white shirts, white dresses, etc. For multiple albedos, we have to tackle the ill-posed problem shown in Equation (6). Before starting this project, we had planned to use other constraints such as a so-called “integrability constraint.” However, we have chosen the smoothness constraint for constraining the problem since the integrability constraint solely cannot solve the problem. Surface normal n can be expressed as the gradients *p* and *q* (Equation (35)). Equation (6) can be rewritten as follows.

(51) $$\begin{array}{ccc} I_{0}\left(x,y\right) & = & f\left(A_{0}\left(x,y\right),p\left(x,y\right),q\left(x,y\right)\right)\;,\\ & \vdots & \\ I_{6}\left(x,y\right) & = & f\left(A_{6}\left(x,y\right),p\left(x,y\right),q\left(x,y\right)\right)\;. \end{array}$$

Namely, we have 9 unknowns ($A_{0}$, …, $A_{6}$, *p*, and *q*) and 7 equations per pixel. Smoothness constraint for *p* and *q* can be represented as follows.

(52) $$\begin{array}{ccc} p(x,y) & = & \frac{1}{4}\left(p(x,y-1)+p(x-1,y)+p(x+1,y)+p(x,y+1)\right)\;,\\ q(x,y) & = & \frac{1}{4}\left(q(x,y-1)+q(x-1,y)+q(x+1,y)+q(x,y+1)\right)\;. \end{array}$$

Since there are additional two constraints per pixel which results in 9 equations per pixel, we can solve the problem. Integrability constraint can be represented as follows.

(53) p(x,y+1)−p(x,y)=q(x+1,y)−q(x,y).

Since only one constraint is added per pixel, we cannot determine 9 parameters from 8 equations. This is the reason why we use smoothness constraint rather than integrability constraint.

The over-smoothing problem is an unavoidable effect in the current approach, which relies on Equation (6). Our future work is to drastically change our approach such that it does not depend on Equation (6). We have to fundamentally consider the basic theory in order to improve the performance of color photometric stereo.

## 5. Conclusions

In this study, surface normal estimation of multicolored objects was conducted by the multi-spectral color photometric stereo method using the median filter and occluding boundary. Note that the conventional color photometric stereo method is an ill-posed problem. Constraining the surface normal and albedo using the median filter sucessfully solved this problem. In addition, we used the approximate shape calculated from the occluding boundary as the initial guess for the surface normal. Finally, we assembled measurement hardware that illuminates the object with seven different spectra and captured the image by a seven-band multispectral camera.

As discussed in Section 4.3, our method faces several problems in terms of both hardware and software. These problems cannot be solved with a minor update, so we need a drastic change for further improvement. In the future, we will disassemble the current measurement hardware and create a more useful system. For example, in order to make the hardware robust to shadow, it is better to add more lights and observe the scene with a multispectral camera with more than 7 channels. The current method used one point light per channel, however, using area light is one choice for improvement in order to avoid the shadows. A polarization filter is also useful to remove the specular reflection. Additional future work is to reconsider the basic theory and fundamentally reorganize the approach of the algorithm. In order to apply the method to non-Lambertian BRDF, it is useful to measure the database of actual object with proposed system and train them using deep learning or other machine learnings. A database of spectral reflectance of various object decreases the number of unknowns which can make the problem well-posed. Using additional sensors such as RGB-D camera is also interesting.

## Figures and Tables

**Table 1:** Peak wavelength and full width at half maximum (FWHM) for each light source.

Light	0	1	2	3	4	5	6
Peak	750 nm	632 nm	610 nm	550 nm	520 nm	470 nm	430 nm
FWHM	10 nm	10 nm	10 nm	10 nm	10 nm	10 nm	10 nm
